# Investigating Mechanically Activated Currents from Trigeminal Neurons of Nonhuman Primates

**DOI:** 10.1523/ENEURO.0054-25.2025

**Published:** 2025-05-09

**Authors:** Karen A. Lindquist, Jennifer M. Mecklenburg, Anahit H. Hovhannisyan, Shivani B. Ruparel, RE-JOIN Consortium Investigators, Armen N. Akopian

**Affiliations:** ^1^Department of Pharmacology and Physiology, School of Medicine, University of Texas Health Science Center at San Antonio, San Antonio, Texas 78229; ^2^Integrated Biomedical Sciences (IBMS) Program, University of Texas Health Science Center at San Antonio, San Antonio, Texas 78229; ^3^Center for Pain Therapeutics and Addiction Research, School of Dentistry, University of Texas Health San Antonio, San Antonio, Texas 78229; ^4^Department of Endodontics, School of Dentistry, University of Texas Health San Antonio, San Antonio, Texas 78229

**Keywords:** mechnoactivated current, nonhuman primates, sensory neurons, trigeminal ganglia

## Abstract

Pain sensation often involves mechanical modalities. Mechanically activated (MA) ion channels on sensory neurons underly responsiveness to mechanical stimuli. MA current properties have mainly been derived from rodent sensory neurons. This study aimed to address gaps in knowledge regarding MA current properties in trigeminal (TG) neurons of a higher-order species, common marmoset nonhuman primates (NHP). MA currents triggered by a piezoactuator were recorded in patch-clamp configuration. MA responses were associated with action potential (AP) properties, such as width, dV/dt on the falling phase, and presence/absence of AP firing in NHP TG neurons. According to responsiveness to mechanical stimuli and AP properties, marmoset TG neurons were clustered into four S-type and five M-type groups. S-type TG neurons had broader AP with two dV/dt peaks on the AP falling phase. Only one S-type group of NHP TG neurons produced small MA currents. M-type TG neurons had narrow AP without two dV/dt peaks on the AP falling phase. M-type NHP TG neurons, except for one group, showed MA currents. We additionally used immunohistochemistry to confirm the presence of known sensory neuronal types such as unmyelinated peptidergic CGRP^+^/trpV1^+^, unmyelinated nonpeptidergic MrgprD^+^ and CGRP^−^/trpV1^+^, and myelinated peptidergic CGRP^+^/trpV1^−^ and nonpeptidergic CGRP^−^ and PV^+^ NHP TG neurons. Overall, marmoset TG neurons and associated MA currents have many similarities compared with reported data from mouse sensory neurons. However, there are notable differences such as lower percentage of small NHP TG neurons responding to mechanical stimuli and absence of fast inactivating MA currents.

## Significance Statement

Understanding the mechanical responses in trigeminal (TG) neurons is pivotal for elucidating the mechanisms of somatosensation and gaining insights into the cellular basis of acute and chronic pain in head and neck area. Identifying specific properties of piezo actuated mechano-activated (MA) currents from non-human primates (NHPs) is of fundamental importance, underscoring the relevance of this study. Based on electrical properties of neurons, nine distinct types of NHP TG neurons were identified. Overall, NHP TG neurons have many similarities with the reported properties of mouse dorsal root ganglion (DRG) and TG neurons. However, there are notable differences such as a low percentage of neurons responding to mechanical stimuli among the smaller TG neurons and an absence of fast inactivating MA currents. 

## Introduction

Pain perception often involves mechanical modalities (Lolignier et al., 2015). Specialized sensory neurons detect and respond to a range of painful and nonpainful mechanical stimuli from the environment. The sensory neuronal cell bodies are in several ganglia, including trigeminal (TG) and dorsal root ganglia (DRG; Hao and Delmas, 2011). Sensory neurons in TG and DRG are neurochemically and functionally diverse (Lumpkin and Caterina, 2007; Usoskin et al., 2015; Uceyler, 2016; LaPaglia et al., 2018; Bhuiyan et al., 2024). Traditionally, they are classified by myelination status and conduction velocity (CV; Basbaum and Braz, 2010; Baumgartner, 2010). They are also categorized based on their functional roles, such as polymodal nociceptors and mechanoheat nociceptors (Djouhri et al., 1998; Djouhri and Lawson, 2004; Basbaum et al., 2009).

A piezoactuator device generates mechanically activated (MA) currents by applying controlled deformation to the plasma membrane, mimicking mechanical indentation observed in vivo (McCarter et al., 1999; Hao and Delmas, 2011). Using this approach, distinct MA currents have been recorded in rodent DRG neurons (Drew et al., 2002; Di Castro et al., 2006; Coste et al., 2010; Dubin et al., 2012; Narayanan et al., 2018; Zhang et al., 2019; Romero et al., 2020, 2023). Previous studies have provided valuable insights; however, several gaps remain. First, most data on MA currents have been gathered from DRG neurons, while TG neuronal MA currents remain largely unexplored. TG neuronal MA currents were mainly recorded from birds (Schneider et al., 2017, 2019; Ziolkowski et al., 2023) and mouse neurons innervating the cornea (Fernandez-Trillo et al., 2020). Second, MA currents in human sensory neurons have been examined using iPSC-derived neurons (Schrenk-Siemens et al., 2015; Romero et al., 2020), which may not fully replicate the properties of mature sensory neurons. Third, PIEZO2 expression levels and in vivo Ca^2+^-imaging–based mechanical responses were observed across various DRG and TG neurons in rodents, primates, and humans (Sharma et al., 2020; von Buchholtz et al., 2020, 2021; Kupari et al., 2021; Bhuiyan et al., 2024; Qi et al., 2024). However, the relationship between MA currents and specific sensory neuron types remains poorly defined. Notable exceptions include studies examining MA currents in skin and muscle-innervating DRG neurons (Weyer et al., 2015) and TG neurons innervating the cornea (Fernandez-Trillo et al., 2020).

Understanding sensory neuronal types based on their electrical and pharmacological properties is vital for elucidating the molecular and cellular basis of mechanical hypersensitivity. Here, we examined MA currents in TG neurons from nonhuman primates (NHP), common marmosets, to provide evolutionarily closer to human data. We also associated MA currents with action potential (AP) properties. This association may not serve as a precise classification of NHP TG sensory neurons. Nevertheless, such a link generated by patch-clamp recording (Petruska et al., 2000; Patil et al., 2018; Lindquist et al., 2021) and in vivo intracellular recording from TG neurons (Boada, 2013; Boada et al., 2014) supplemented transcriptomic and anatomical data for a more precise classification of sensory neurons.

## Materials and Methods

### Ethical approval

This study adheres to the ARRIVE 2.0 guidelines (Percie du Sert et al., 2020). All animal care and experimental procedures complied with the United States Public Health Service Policy on Humane Care and Use of Laboratory Animals, the *Guide for the Care and Use of Laboratory Animals*, and the American Society of Primatologists’ principles for the ethical treatment of NHP. We followed guidelines from the National Institutes of Health (NIH) and the Society for Neuroscience (SfN) to minimize the number of animals used and their suffering. All procedures were approved by the Institutional Animal Care and Use Committee (IACUC) of the University of Texas Health Science Center at San Antonio (UTHSCSA) and the Texas Biomedical Research Institute (TBRI). The IACUC protocol title is “Plasticity of lymphotoxin-beta signaling and orofacial pain in non-human primates” (UTHSCSA: 20200021AR; TBRI: 1821 CJ 0).

### Animal subjects, tissue collection, and transfer

Based on the availability of mainly male NHP, we collected tissues from five adult male common marmosets (*Callithrix jacchus*), aged 3–5 years. Tissue samples were obtained at necropsy from the UTHSCSA and TBRI “Tissue Share” program, where animals were killed under IACUC-approved endpoints. None of the animals had head or neck injuries or systemic infections. Following euthanasia by veterinary staff at either UTHSCSA or TBRI, trigeminal ganglia (TG) were collected within 2 h of death. Collected tissues were placed in Hank's solution on ice for patch-clamp electrophysiology or fixed in 4% paraformaldehyde (PFA) for immunohistochemistry (IHC; Ibrahim et al., 2023). Collected samples into Hank's or 4% paraformaldehyde (PFA) solution were transported by car (15 min drive) from TBRI to UTHSCSA.

### Primary trigeminal ganglion neuronal culture

TG cultures were initiated immediately upon sample arrival. Two TG were incubated 1 ml of Hank's solution containing 30 µl of 50 mg/ml collagenase I (Worthington, code #CLS-1, 230 U/mg) and 7.5 µl of 50 mg/ml dispase (Roche, catalog #4942078001) for 45 min at 37°C in water bath. Tissues were washed twice with DMEM containing 5% fetal bovine serum, 2 mM ʟ-glutamine, 100 U/ml penicillin, and 100 µg/ml streptomycin and centrifuged at 1,000 rpm for 75 s. TG cells were mechanically dissociated in 1 ml of DMEM with 5% fetal bovine serum, 2 mM ʟ-glutamine, 100 U/ml penicillin, and 100 µg/ml streptomycin. Mechanical dissociation was carried out with 1 ml pipette using 15–20 strokes. Dissociated TG cells were directly (without filtering) plated on 12 mm German glass coverslips coated with laminin and poly-d-lysine (Corning, catalog #08-774-385). No growth factors were added to the media, since growth factors like NGF are known to sensitize sensory neurons leading to enhancement of MA currents and unmasking silent nociceptors (Ritter and Mendell, 1992; Price et al., 2005; Prato et al., 2017; Schaefer et al., 2018; Shrivastava et al., 2021). Electrophysiological experiments were conducted 12–36 h after plating.

### Whole-cell patch-clamp electrophysiology

Recordings were performed at 22–24°C using an Axopatch 200B amplifier and analyzed with Axon pCLAMP 11.2 software (Molecular Devices). The recording configuration was “whole-cell *β* = 1.” Data were filtered at 0.5–5 kHz and sampled at 2–20 kHz depending on current kinetics. Fire-polished glass electrodes (2–6 MΩ resistance) were used. Access resistance (Rs) was compensated to achieve 4–8 MΩ. Data were discarded if resting membrane potential (RMP) was less than −35 mV (Djouhri et al., 1998; Fang et al., 2005), Rs changed to >20% during recording, leak currents were >100 pA, or input resistance was <100 MΩ, which could be an indication of leak. Currents were considered positive when amplitudes were at least fivefold larger than noise levels. The standard external solution contained 140 mM NaCl, 5 mM KCl, 2 mM CaCl_2_, 1 mM MgCl_2_, 10 mM d-glucose, and 10 mM HEPES (pH 7.4). The standard pipette solution (SIS) contained 140 mM KCl, 1 mM MgCl_2_, 1 mM CaCl_2_, 10 mM EGTA, 10 mM d-glucose, and 10 mM HEPES (pH 7.3) along with 2.5 mM ATP and 0.2 mM GTP. The osmotic pressure of the external and internal solutions could affect the amplitude and inactivation kinetics of the MA current. Hence, we constantly kept 310 mOsm for the external solution and 290 mOsm for the pipette solution. Osmolarity was measured on a 5520 Vapor Pressure Osmometer (Wescor) and adjusted by water or mannitol.

### Electrophysiology protocols, MA current recording, and data analysis

After patching a selected neuron, recordings were made using a sequence of protocols. (1) In the current-clamp configuration, a single AP response was generated with a 1, 2, 3, 4, or 5 nA (separate sweeps) 0.5 ms current pulse (Petruska et al., 2000; Patil et al., 2018; Lindquist et al., 2021). (2) Following this, AP trains were elicited by applying step currents of 50–550 pA with 100 pA increments for 1-s-long sweeps or 200–2,000 pA with 300 pA increments for the few >80–100 pF neurons. (3) Electronics were then switched to voltage-clamp configuration (*V*_hold_ at −60 mV), and mechanically evoked (MA) currents were recorded (elaborated below). The following variables were measured: cell capacitance (C_m_ in pF), resting membrane potential (RMP in mV), AP width at an RMP level (dB in ms), dV/dt on a downward slope of AP, afterhyperpolarization (AHP) peak (in mV), AHP_80_ duration (in ms), threshold of activation for MA currents (in actuating pipette displacement μm), their peak current densities, and *τ* (time constant in ms) decay for MA current inactivation kinetics using the equation *I* = ΔI*exp^(−t/*τ*_inact_) (Petruska et al., 2000). Fitting and decay tau (*τ*; ms) calculation was performed using pCLAMP 11.2 software (Molecular Devices).

In a voltage-clamp (*V*_hold_ = −60 mV) configuration, MA currents were induced by mechanical stimulation directly applied by a fire-polished blunt borosilicate glass pipette (BF100-50-10, Sutter Instrument) with a tip diameter of 1–3 µm, held at a 45° angle from the coverslip. This pipette was driven by a piezoelectric actuator (P-841.6, Physik Instrumente; McCarter et al., 1999; Hao and Delmas, 2011). Probe movement was controlled by a Digital Piezo Controller (E-709, Physik Instrumente), which in turn was under the control of Axopatch 200B amplifier and Clampex 11.2 software (Molecular Devices). The tip was advanced in increasing 1.5 µm displacements, each held for 300 ms, with 10 s between steps to avoid sensitization or desensitization of currents during 10 consecutive sweeps (Weyer et al., 2015). If mechanically responsive, current amplitudes incrementally increased as each mechanical poke deepened (Rugiero et al., 2010).

### Immunohistochemistry

Immunostaining of marmoset TGs was performed as described previously (Hovhannisyan et al., 2023; Tram et al., 2023). TG tissues were fixed in 4% PFA for at least 16 h, cryoprotected with 10 and 30% sucrose in PBS for at least 24 h, embedded in Neg-50 (Richard Allan Scientific), and cryosectioned at 20 μm thickness. Sections were blocked with 4% normal donkey serum (Sigma-Aldrich), 2% bovine gamma globulin (Sigma-Aldrich), and 0.3% Triton X-100 (Thermo Fisher Scientific) in 0.1 M PBS for 90 min at room temperature (RT) and subsequently incubated with primary antibodies for 24–36 h. Sections were then washed with PBS from unbound primary antibodies, blocked, and incubated for 90 min at RT with appropriate fluorophore-conjugated secondary antibodies (Jackson ImmunoResearch). Finally, tissue sections were washed three times for 5 min with 0.1 M PBS and two times for 5 min in diH_2_O, air-dried, and covered with Vectashield Antifade Mounting Medium (VectorLabs). The following previously characterized primary antibodies were used: anti-CGRP guinea pig polyclonal (Synaptic Systems; catalog #414 004; 1:200; Samms et al., 2022); anti-TRPV1 rabbit polyclonal (Novus Biologicals; catalog #NBP1-71774SS; 1:200; Moutafidi et al., 2021); anti-mrgprD rabbit polyclonal (Alomone Labs; catalog #ASR-031; 1:200; de Carvalho Santuchi et al., 2019; Lindquist et al., 2021); anti-tyrosine hydroxylase (TH) chicken polyclonal (Neuromics; catalog #CH23006; 1:300; Ferreira-Pinto et al., 2021); and anti-parvalbumin (PV) rabbit polyclonal (Novus Biologicals; catalog #NB120-11427SS; 1:200; Lewis et al., 2021).

*Z*-stack images were captured with a Keyence BZ-X810 all-in-one microscope under the “sectioning” mode using a 2×, 10×, or 20× objective. Control IHC was performed on tissue sections processed as described but either lacking primary antibodies or lacking primary and secondary antibodies. Settings were determined in such a way that no-primary antibodies and both no-primary and no-secondary antibody controls did not show any positive signal. Then, images were taken using these fixed acquisition parameters across all groups. For cell counting, *Z*-stack IHC images with 10× or 20× objectives were obtained from 3–5 independent tissue sections from 2–3 primates/isolations. Counted marker-positive neurons were presented as percentages of the total neuron numbers. All TG could have been labeled by pan-sensory neuronal marker (NeuN; Wu et al., 2018; Mecklenburg et al., 2023). However, NeuN did not clearly label at least 25% of NHP TG neurons, and using polyclonal rabbit-made NeuN could compromise the usage of many available antibodies. Another pan-sensory neuronal marker β-III tubulin also did not label a portion of TG neurons, while Protein Gene Product 9.5 (PGP 9.5) antibodies, which effectively label all sensory neurites in tissues, are not effective for labeling sensory neuronal cell bodies (Hovhannisyan et al., 2023). Accordingly, the total neuronal numbers were estimated by taking section pictures with high gain and highlighting autofluorescence in all TG neurons. Nonneuronal cells displayed autofluorescence as well. However, they are morphologically distinct from sensory neurons. Neurons were counted using ImageJ software (Tram et al., 2023). The mean values from counts across 3–5 sections generated from an NHP TG represented data for a biological replicate. Thus, *n* = 3 represents three NHP TG as the biological replicates.

### Statistical analysis

GraphPad Prism 10 (GraphPad) was used for statistical analyses. Data in the figures are means ± SEM, with *n* referring to the number of sectioned TG for IHC and the number of analyzed recorded cells for electrophysiology. Differences between expression percentages of markers in TG and electrophysiological parameters between groups were assessed by unpaired *t* test or one-way ANOVA with Tukey's post hoc tests, and each column was compared with all other columns. A difference is accepted as statistically significant when *p* < 0.05. Interaction *F* ratios and the associated *p*-values are reported.

## Results

### Electrical and MA current parameters for grouping NHP TG neurons

Sensory neurons could be grouped by clustering using multiple parameters, including transcriptomic profiles (Usoskin et al., 2015; Yang et al., 2022; Qi et al., 2024), expressions of markers (Hovhannisyan et al., 2023; Qi et al., 2024), ex vivo or in vivo extracellular recordings (Djouhri et al., 1998; Djouhri and Lawson, 2004; Boada, 2013), electrophysiological properties derived from patch-clamp recordings (Petruska et al., 2000; Patil et al., 2018; Lindquist et al., 2021), or a combination of these methods (Zheng et al., 2019; Qi et al., 2024). In rodent models, sensory neuron size is a dependable indicator for differentiating between DRG C- and A-fiber neurons (Villiere and McLachlan, 1996; Patil et al., 2018; Lindquist et al., 2021). However, in NHP TG neurons, this distinction based on size (capacitance) was less clear (Table 1). Thus, many NHP TG neurons exceeded a capacitance of 100 pF. Hence, NHP TG cell sizes are not the best clustering parameter.

**Table 1.** Electrophysiological properties of common marmoset TG neuronal groups

Group	*N*	Cap (pF)	RMP (mV)	dB (ms)	dV/dt^a^ (mV)	AHP_80_ (ms)	AHP peak (mV)	AP Thresh (pA)	AP Train (Hz)	MA (Y/N)
S1	27	68.9 ± 4.9	−50 ± 1.7	10.2 ± 0.6	−12.3 ± 1.2	28.7 ± 5.8	−9.3 ± 0.8	N/A	N/A ^c^	N
S2	29	62.6 ± 3.7	−56.4 ± 1.7	8.1 ± 0.3	−12.6 ± 1.7	48.1 ± 7.5	−11.4 ± 0.7	125.9 ± 12.8	8.4 ± 1.8	N
S3	12	59.1 ± 5.6	−63.4 ± 3.5	10.7 ± 1.1	−13.2 ± 1.4	32.7 ± 9.1	−7.5 ± 1	161.1 ± 20	4.6 ± 0.8	Y
S4	15	65.3 ± 6.2	−38.3 ± 2.4	7.6 ± 0.9	Linear ^b^	8 ± 3.1	−5.5 ± 0.8	N/A	N/A ^d^	N
M1	32	88.8 ± 4.1	−62.8 ± 1.5	5.1 ± 0.3	−0.6 ± 1.3	48.7 ± 7.1	−10.3 ± 0.9	173 ± 28.1	8.4 ± 2.7	N
M2	21	104.4 ± 3.2	−63.6 ± 1.5	4.2 ± 0.4	0.3 ± 1.7	55 ± 11.3	−9.6 ± 1.2	175 ± 25	5.5 ± 1.7	Y
M3	9	80.9 ± 7.6	−68.3 ± 1.9	2.6 ± 0.1	Linear ^b^	1.7 ± 0.9	−4 ± 0.4	N/A	N/A ^d^	Y
M4	15	81.7 ± 6.4	−64.4 ± 1	2.6 ± 0.1	Linear ^b^	41.9 ± 10.6	−7.8 ± 0.9	N/A	N/A ^d^	Y
M5	25	72.9 ± 4.8	−59 ± 2.3	2.3 ± 0.1	Linear ^b^	11.8 ± 1.9	−9.4 ± 0.9	N/A	N/A ^d^	Y

**Figure 1.**
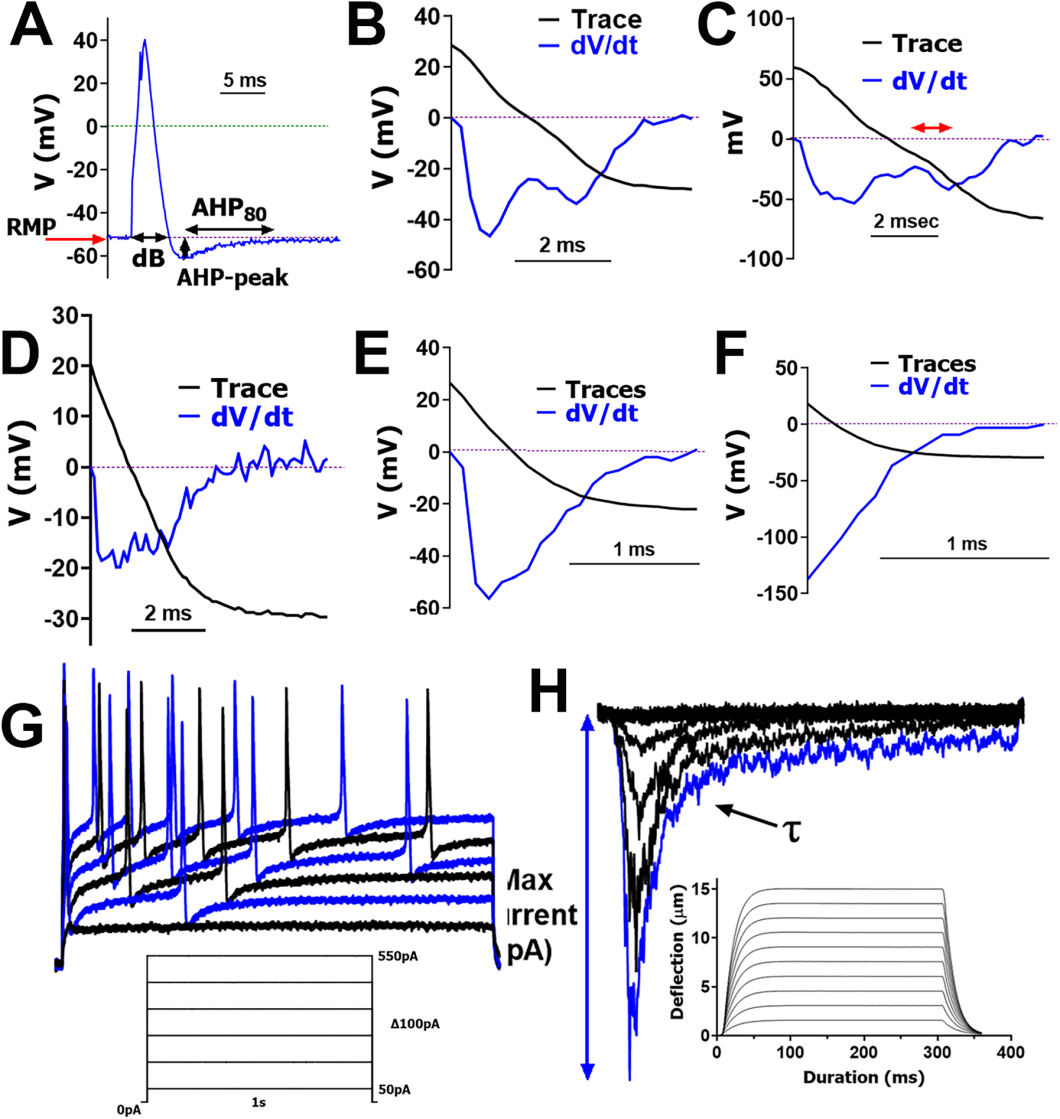
Electrophysiology protocols. ***A***, A single action potential (AP) is elicited in an NHP TG neuron by a brief current injection of 1 nA for 0.5 ms. We analyzed indicated AP and afterhyperpolarization (AHP) parameters, including resting membrane potential (RMP), duration at base (dB), magnitude of AHP peak, and the time required for the AHP (measured in ms) to decay by 80% (AHP_80_). ***B***, ***C***, dV/dt for the falling phase of broad (dB > 5 ms) AP with a characteristic “hump” or “bow.” ***D***, dV/dt for the falling phase of AP (dB < 5 ms) with a characteristic slight “deflection.” ***E***, ***F***, dV/dt for the falling phase of fast AP (dB < 4 ms) with a “straight” falling phase. ***G***, AP trains are triggered by applying steps of increasing current injections, 50–550 pA with 100 pA increments. A schematic of the protocol used, and a sample recording is shown. ***H***, MA currents are activated by a piezoactuator controlled by a piezoelectric device. A graphical representation of actuator movement is shown. Each poke extends by 1.5 µm increments and is held for 300 ms before returning to the starting position for 10 s of relaxation. A total of 10 progressively deeper pokes are administered, each increasing by an additional 15 µm with the final poke going in 15 µm deep. Sample recording from an NHP TG neuron is shown; *τ* is indicated.

Values are means ± SEM.

a Distance between lowest points between two dV/dt peaks and the second dV/dt peak (Fig. 1*C*, red arrow).

b There is no second dV/dt peak.

c One neuron from the group fired two APs.

d Two neurons fired small trains of APs.

**Figure 2.**
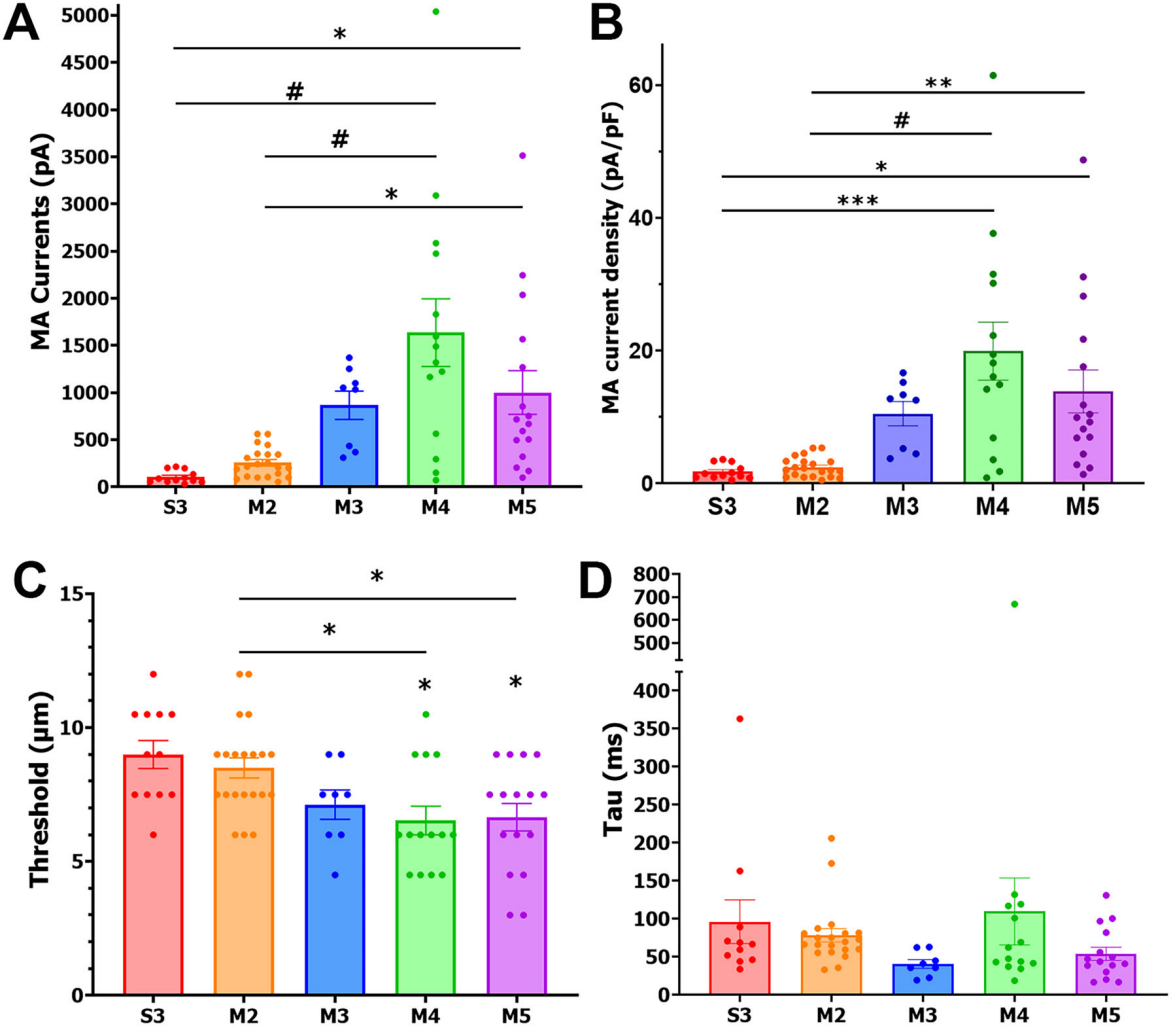
MA currents from NHP TG neurons. Five NHP neuronal subgroups (labeled under the *x*-axis) responded to mechanical stimulation. These neuronal groups showed different characteristics. ***A***, Max MA current amplitudes (pA) from these five MA groups of NHP TG neurons. ***B***, Max MA current density (pA/pF) from these MA groups of NHP TG neurons. ***C***, Activation threshold in actuator distance (μm) traveled for these NHP TG neuronal subgroups. ***D***, Decay kinetics (ms) of MA currents for these NHP TG neuronal subgroups. Data were analyzed by one-way ANOVA in each column compared with others followed by Bonferroni's post hoc tests; *p < 0.05; **p < 0.01; ***p < 0.001; ^#^*p* < 0.0001.

We classified NHP TG neurons based on action potential (AP) parameters obtained via patch-clamp recordings for TG neurons responding and nonresponding to mechanical piezoactuating stimulation. AP's duration at base (dB) recorded by patch-clamp or intracellular recording with sharp electrodes is a reliable parameter for grouping of sensory neurons (Petruska et al., 2000; Boada, 2013; Boada et al., 2014; Zheng et al., 2019; Lindquist et al., 2021; Fig. 1*A*). Another suitable parameter for clustering is dV/dt for the downward slope of the AP. Some neurons with broad APs [duration at base (dB), >5–6 ms] had a characteristic “hump” on the downward slope of the AP (Patil et al., 2018; Lindquist et al., 2021). These neurons have a dV/dt that exhibited two peaks at negative voltages (Fig. 1*B*,*C*). The size of the second peaks relative to the lowest point between the two peaks could be quantified (Fig. 1*C*, red arrow; Table 1). TG neurons with an AP duration of 2–5 ms had a distinct “deflection” on the downward slope (Patil et al., 2018; Lindquist et al., 2021). The dV/dt of these neurons did not have a second peak but showed a broad first peak (Fig. 1*D*). Neurons with fast APs (duration, 1–3 ms) and a straight downward slope had a dV/dt without a second peak (Fig. 1*E*,*F*). Afterhyperpolarization (AHP) peak and AHP recovery time (AHP_80_) are presented for each cluster, but variability in these parameters made them unsuitable for clustering (Fig. 1*A*). We also evaluated whether TG neurons were capable of producing evoked AP train (Fig. 1*G*). Finally, neurons were also classified as mechanically sensitive and insensitive (Fig. 1*G*). Magnitudes of MA currents (Fig. 2*A*), current density (Fig. 2*B*), and activation thresholds (Fig. 2*C*) were suitable and reliable parameters for grouping TG neurons (Table 2). However, we did not classify MA currents based on their kinetics (rapid, intermediate, or slow; McCarter et al., 1999; Coste et al., 2010; Hao and Delmas, 2010), as no significant differences were observed in the decay kinetics between the MA currents between groups (one-way ANOVA; *F*_(4, 64)_ = 1.216; *p* = 0.3126; Fig. 2*C*; *n* *=* *8-21*; Table 2; Fig. 2*D*; see Discussion).

**Table 2.** Properties of MA currents

Group	MA threshold (µm)	MA max current (pA)	MA current density (pA/pF)	MA *τ* (ms)
S3	24 ± 1.4	105.8 ± 18.8	1.8 ± 0.3	96.2 ± 28.7
M2	22.7 ± 1	257 ± 34	2.5 ± 0.3	78.4 ± 8.8
M3	19 ± 1.5	865.6 ± 150.2	10.5 ± 1.8	40.7 ± 5.7
M4	17.4 ± 1.4	1,636 ± 359	19.95 ± 4.38	109.8 ± 44.1
M5	17.8 ± 1.4	1,001 ± 231.5	13.9 ± 3.2	54 ± 8.7

Values are means ± SEM. MA current parameters are presented. MA threshold refers to the displacement depth at which neurons first start responding to mechanical stimulation by sustaining inward currents. MA max current refers to the peak current amplitude of the inward currents elicited by mechanical stimulation. MA *τ* refers to the decay kinetics of MA currents. They were not separated into decay kinetic subtypes.

### Groups of mechanosensitive and mechanoinsensitive common marmoset TG neurons

More than 200 NHP TG neurons were recorded from five NHP-generated five cultures. The 185 common marmoset TG neurons that passed quality control were included in the analysis. Of the recorded neurons, 72 out of 185 responded to mechanical stimulation, which is consistent with previously reported proportions for mouse TG neurons innervating the cornea (Fernandez-Trillo et al., 2020).

**Figure 3.**
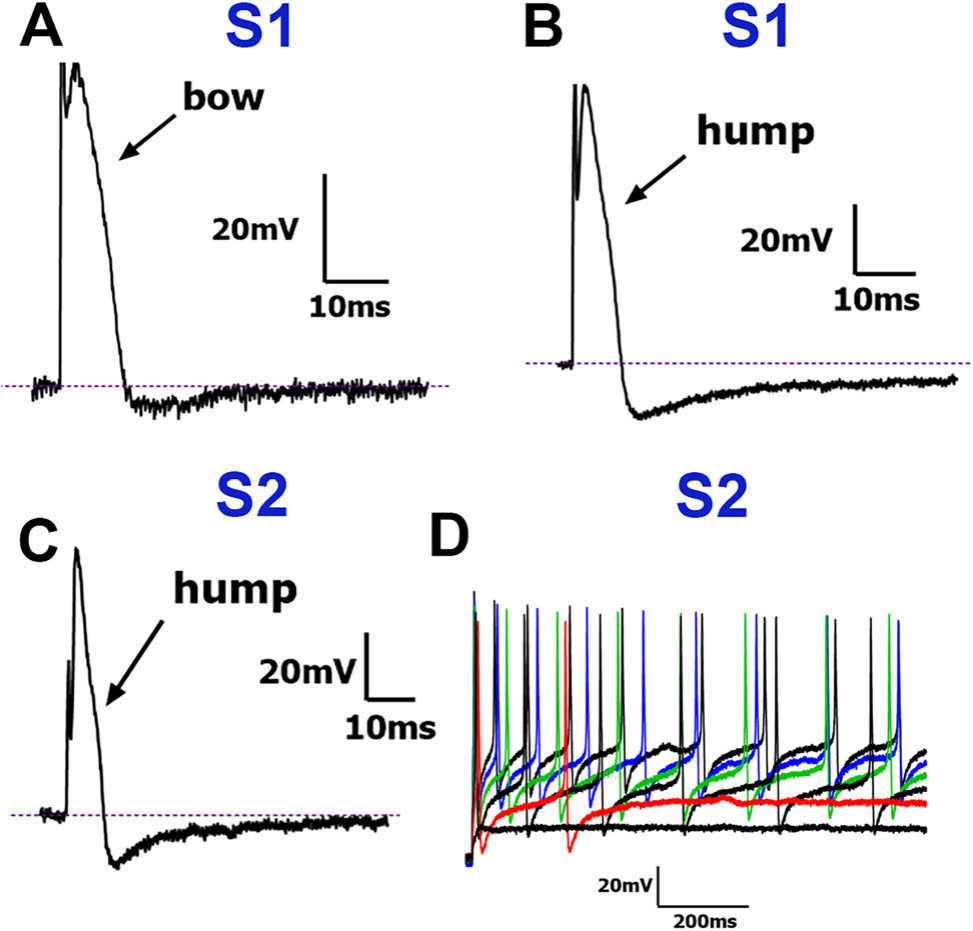
Traces representing current signatures from S1 and S2 NHP TG neuronal groups. ***A***, Representative AP trace with “bow” on the AP falling phase belonging to the S1 group. ***B***, Representative AP trace with “hump” on the AP falling phase belonging to the S1 group. ***C***, Representative AP with “hump” on the AP falling phase belonging to the S2 group. ***D***, Representative AP train belonging to the S2 group. A characteristic “hump” or “bow” on the downward portion of the AP is indicated with a black arrow in panels ***A***, ***B***, and ***C***. Neuronal groups are specified above traces. Scale bars are presented for each panel.

S1 (Small-1) neurons: The S1 group neurons exhibited long-duration APs (i.e., dB), which were always >5 ms (Table 1). Seven S1 neurons exhibited “bow”-shaped APs on the downward phase (Fig. 3*A*), while the remaining S1 neurons had a characteristic “hump” on the falling phase (Fig. 3*B*). dV/dt curves were similar for neurons showing a “bow” or “hump” on the falling phase of APs and had two peaks (Fig. 1*B*,*C*; Table 1). These neurons did not fire trains of APs nor respond to mechanical stimulation. S1 neurons had the largest soma size among S-type neurons, as indicated by capacitance measurements (Table 1).

S2 (Small-2) neurons: The S2 group neurons showed >5 ms dB and were the most abundant among S-type TG neurons (Table 1). Like S1 neurons, the S2 group had two large dV/dt peaks representing “humps” on the falling phase of AP (Fig. 3*C*; Table 1). These neurons consistently fired AP trains and had the lowest AP firing threshold (Fig. 3*D*, Table 1). The S2 group did not respond to mechanical stimulation.

**Figure 4.**
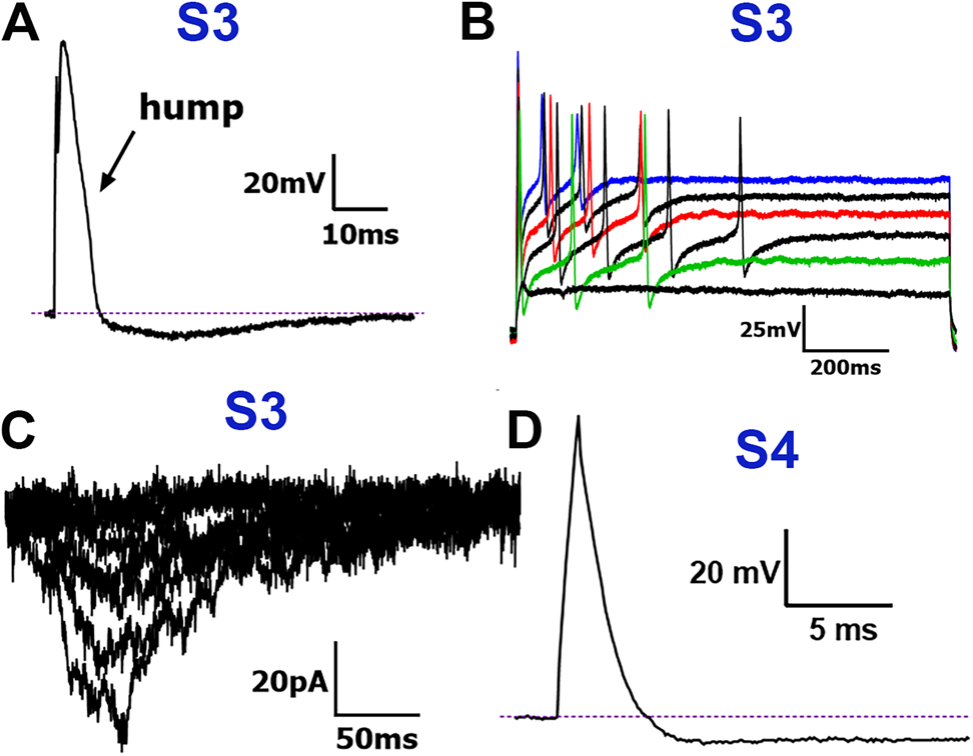
Traces representing current signatures from the S3 and S4 NHP TG neuronal groups. ***A***, Representative AP trace with “hump” on the AP falling phase belonging to the S3 group. ***B***, Representative AP train belonging to the S3 group. ***C***, Representative MA currents from a neuron belonging to the S3 group. ***D***, Representative AP trace belonging to the S4 group. Neuronal groups are specified above traces. Scale bars are presented for each panel.

S3 (Small-3) neurons: S3 neurons, which also had >5 ms dB and two large dV/dt peaks representing characteristic “hump” (Fig. 4*A*), fired at least one AP in train (Fig. 4*B*). The crucial distinction was that S3 was the only S-type subgroup that exhibited MA currents (Fig. 4*C*). Compared to the other MA current-exhibiting subgroups, S3 neurons had the highest activation threshold and produced the smallest MA current amplitudes, though these values were not significantly different from those of M2 neurons (one-way ANOVA; *F*_(4,66)_ = 9.596; *n* = 12 and 21; Table 2, Fig. 2*A*–*C*).

S4 (Small-4) neurons: The S4 group was the only S-type subgroup with long-duration APs (>4 ms dB) that lacked double dV/dt peaks (Fig. 4*D*; Table 1). These neurons had the smallest AP dB among S-type neurons, though still longer than that of M-type neurons (Table 1). They also exhibited the fastest AHP_80_ times and smallest AHP peak sizes of all S-type neurons (Table 1). S4 neurons did not fire AP trains or respond to mechanical stimuli and had the most depolarized RMP (Table 1).

**Figure 5.**
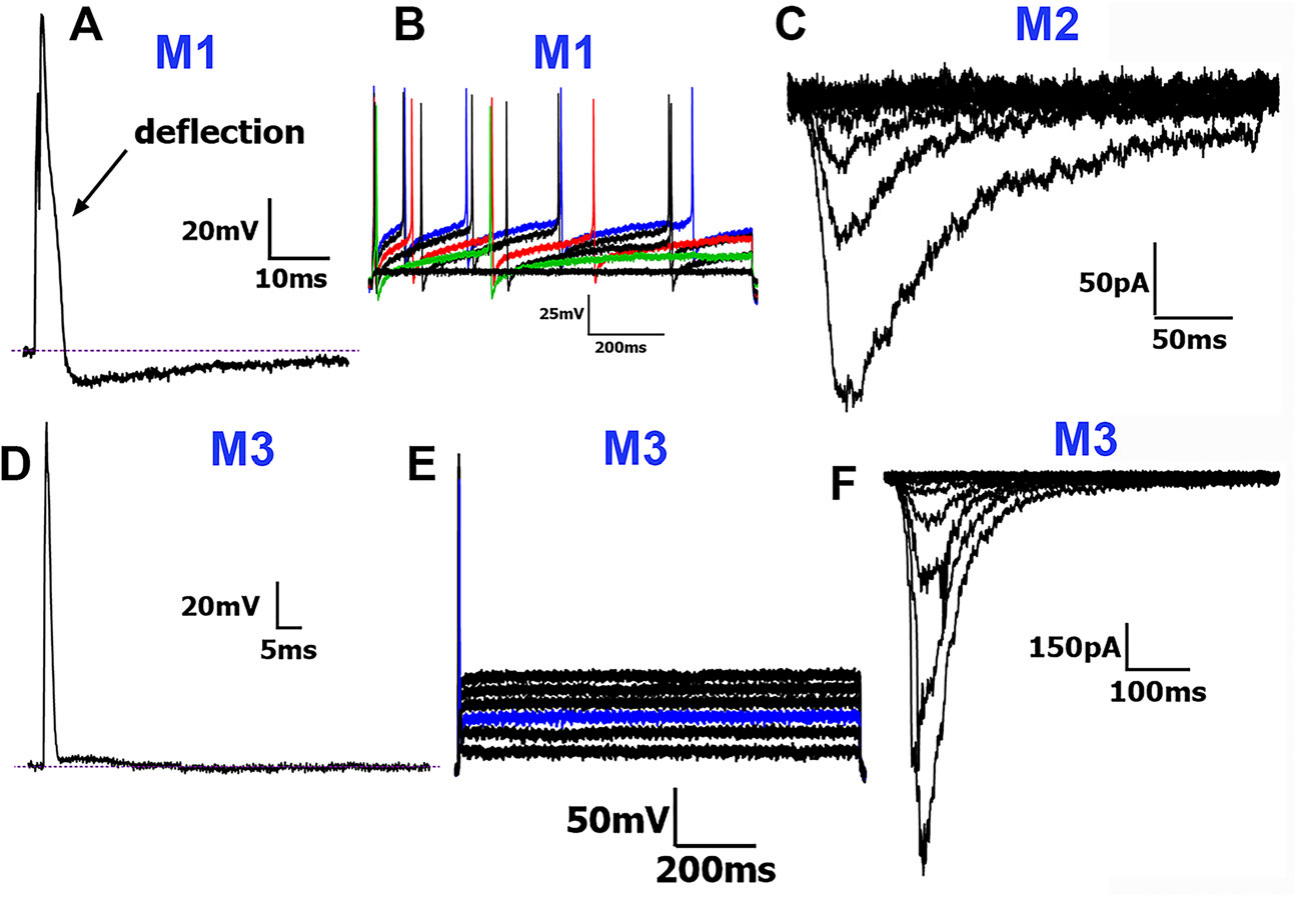
Traces representing current signatures from M1, M2, and M3 NHP TG neuronal groups. ***A***, Representative AP trace with “deflection” on the AP falling phase belonging to the M1 group. The “deflection” is indicated. ***B***, Representative AP train belonging to the M1 group. ***C***, Representative MA currents belonging to the M2 group. ***D***, Representative AP trace belonging to the M3 group. The absence of a true AHP peak (Fig. 1*A*) is observed. ***E***, Representative AP train belonging to the M3 group. ***F***, Representative MA currents belonging to the M3 group. Neuronal groups are specified above traces. Scale bars are presented for each panel.

M1 (Medium-1) neurons: The M1 group exhibited intermediate-duration APs (dB < 6 ms; Table 1). The double peak for dV/dt was not pronounced and often looked like a wide and flat single peak (Fig. 1*D*). Hence, differences between the lowest and highest points of these dV/dt wide and flat peaks were −0.6 ± 1.3 mV. These wide and flat dV/dt peaks reflected the presence of a “deflection” on the falling phase of AP (Fig. 5*A*). M1 neurons were the only M-type neurons that did not respond to mechanical stimulation. Approximately 35% (13/32) of M1 neurons fired AP trains (Fig. 5*B*, Table 1).

M2 (Medium-2) neurons: M2 group neurons were the largest (Table 1). A key difference between M1 and M2 neurons was that M2 neurons showed MA currents (Fig. 5*C*). Otherwise, M1 and M2 neurons had nearly identical AP characteristics and capabilities to fire AP trains (Table 1). Thus, M2 and M1 neurons had similar dB (<6 ms) and dV/dt curves representing a “deflection” on the falling phase of AP (Table 1). One of 21 M2 neurons fired AP trains. Among the M-type groups, M2 neurons had the highest activation thresholds for MA currents and produced the smallest amplitude currents (Figs. 2*A*,*B*, *5C*), with no statistically significant differences in MA characteristics from the S3 group (one-way ANOVA; *F*_(4,66)_ = 9.596; *n* = 12 and 21; Table 2, Fig. 2*A*–*D*).

M3 (Medium-3) neurons: M3 neurons were grouped on the bases of a distinct AHP phase shape, which was without a notable AP undershoot, and their AHP_80_ and AHP peaks were the shortest among all nine groups (Table 1). The M3 group was characterized by fast-duration APs (dB < 4 ms) that lacked a broad or double dV/dt peak for the falling phase of APs seen in S-type neurons and M1 and M2 groups (Fig. 5*D*). Apart from one neuron that fired two APs, A3 neurons did not fire AP trains (Table 1, Fig. 5*E*). Of the fast-duration AP groups (M3–M5), M3 neurons exhibited the smallest amplitudes for MA currents and the fastest decay kinetics (Table 2; Figs. 2, 5*F*).

**Figure 6.**
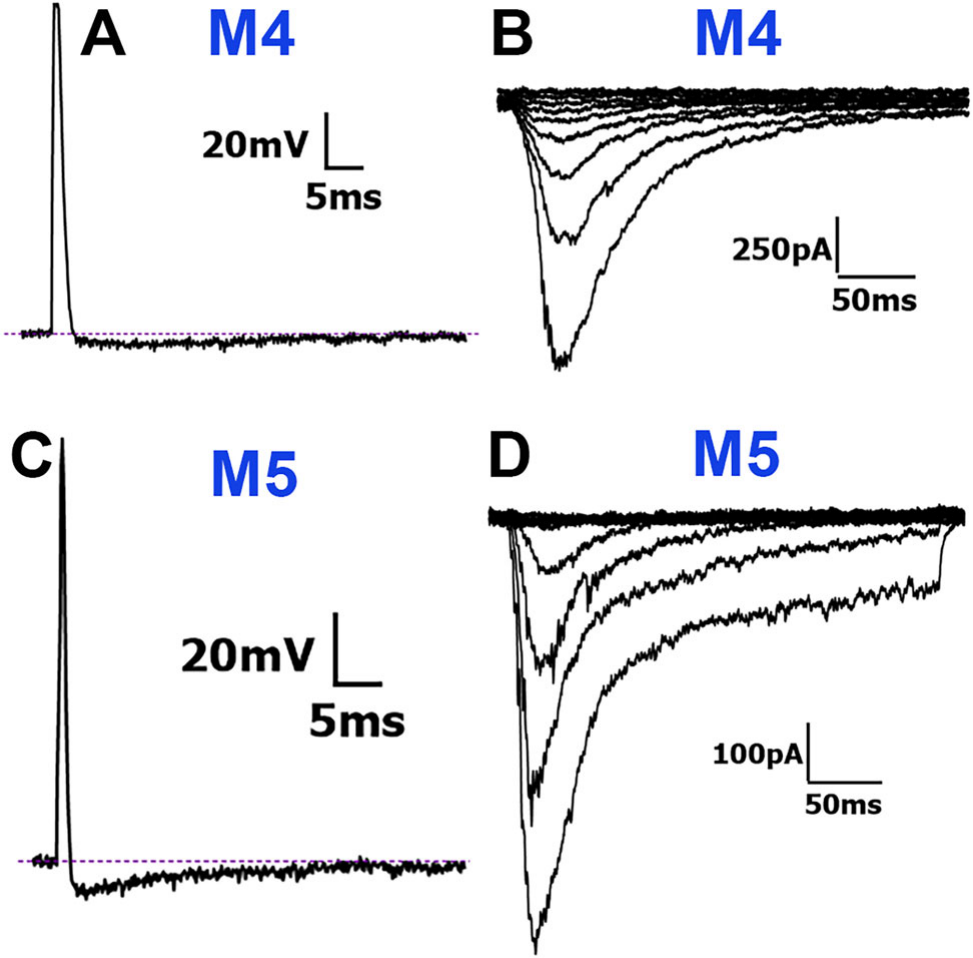
Traces representing current signatures from M4 and M5 NHP TG neuronal groups. ***A***, Representative AP trace belonging to the M4 group. ***B***, Representative MA currents belonging to the M4 group. ***C***, Representative AP trace belonging to the M5 group. ***D***, Representative MA currents belonging to the A5 group. Neuronal groups are specified above traces. Scale bars are presented for each panel.

M4 (Medium-4) neurons: M4 neurons exhibited fast-duration APs (dB < 4 ms) with slower AHPs (AHP_80_ > 20 ms; Fig. 6*A*). Only one M4 neuron fired two APs, while the others did not fire AP trains (Table 1). M4 neurons had the largest MA current amplitudes and the slowest decay kinetics of all MA-exhibiting neurons (Table 2; Figs. *2A*,*C*, 6*B*).

M5 (Medium-5) neurons: The main distinction between M4 and M5 was that M5 neurons had faster AHPs (cutoff for AHP_80_ < 20 ms; Fig. 6*C*). Like M4, M5 neurons exhibited fast-duration APs (dB < 4 ms) but had notable AHP peaks (Fig. 6*C*, Table 1). Apart from two neurons that fired four and seven APs, respectively, A5 neurons did not exhibit AP trains (Table 1). The MA currents recorded from M5 neurons were similar to those recorded from M3 neurons (one-way ANOVA; *F*_(4,66)_ = 9.596; *n* = 8 and 16; Table 2, Fig. 2). MA currents in M4 and M5 neurons had the lowest activation thresholds and higher peak current amplitudes, compared with S3 and M2 neurons, which exhibited high activation thresholds, smaller peak current amplitudes and current density (one-way ANOVA; *F*_(4, 66)_ = 9.596, *p* < 0.0001 for MA current size; *F*_(4, 66)_ = 9.550, *p* < 0.0001 for MA current density; and *F*_(4, 66)_ = 5.240; *p* = 0.001 for MA current threshold; *n* = 8–21; Table 2, Fig. 2*A*–*C*).

In summary, using the patch-clamp electrophysiological classification techniques, we identified four distinct S-type neuronal groups and five M-type neuronal groups for NHP TG neurons. A majority of S-type neurons had no or small MA currents and were associated with broad APs and nonlinear dV/dt for the falling phase of AP, while M-type neurons with the exception of the M1 group exhibited MA current and were linked with faster AP and linear dV/dt (Table 1).

### Expressions of sensory neuronal markers in TG of common marmosets

**Figure 7.**
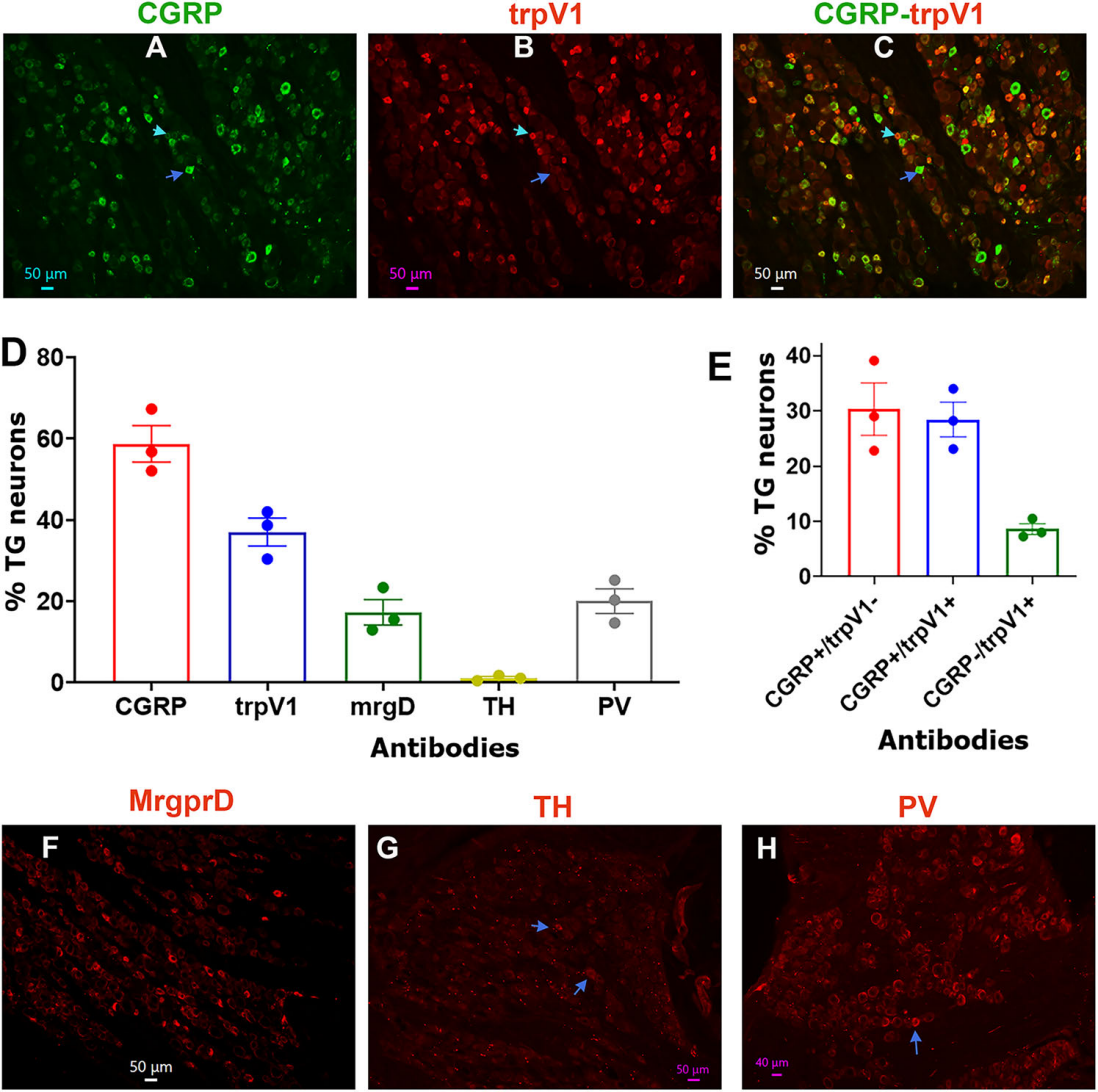
Representation of marker-positive neurons in TG of adult male marmosets. Representative microphotographs show expression patterns for CGRP (***A***), trpV1 (***B***), CGRP + trpV1 (***C***), MrgprD (***F***), tyrosine hydroxylase (TH; ***G***), and parvalbumin (PV; ***H***), in TG of adult male marmosets. The blue arrows on *panels **A***–***C*** indicate CGRP*^+^*/trpV1*^−^* neurons. Cyan arrows on panels ***A***–***C*** show CGRP*^−^*/trpV1*^+^* neurons. The blue arrows on panels ***G*** and ***H*** point to TH- and PV-positive cells, respectively. Antibodies used and matching colors are indicated. Scales are presented in each microphotograph. ***D***, Bar graphs reflect percentages of marker-positive sensory neurons in TG of adult male marmosets. ***E***, Bar graphs reflect relative percentages of CGRP^+^ (peptidergic) and trpV1^+^ neurons in TG of adult male marmosets. The *x*-axis denotes antibodies for markers. *N* = 3.

We next examined representations of neuronal clusters by IHC using sensory neuronal markers. Peptidergic TG neurons were identified by CGRP (Fig. 7*A*). A portion of TrpV1^+^ peptidergic neurons were identified using TRPV1 antibodies (Fig. 7*B*). We found that 58.7 ± 4.5% of neurons were peptidergic, while 37 ± 3.4% of neurons were TrpV1^+^ (Fig. 7*A*–*D*). Among these, CGRP^+^/trpV1^−^ neurons accounted for 30.3 ± 4.8% of all neurons, while CGRP^−^/trpV1^+^ neurons constituted 8.6 ± 1% (Fig. 7*C*,*E*). Additionally, a subset of peptidergic nonmyelinated neurons, which were CGRP^+^/trpV1^+^, comprised 28.4 ± 3.2% of the total population (Fig. 7*C*,*E*). A subset of nonpeptidergic and MrgprD^+^ neurons were detected with MrgprD antibodies in 7.3 ± 3.1% of NHP TG neurons (Fig. 7*D*,*F*). Tyrosine hydroxylase (TH), a marker for sympathetic fibers in the facial muscles of NHP (Hovhannisyan et al., 2023), which is also expressed in C-LTMR DRG neurons (Usoskin et al., 2015), was found to label only a few neurons in NHP TG (Fig. 7*D*,*G*). A-LTMR neurons are typically identified by markers like TrkB, TrkC, calbindin (Calb1), and parvalbumin (PV; Usoskin et al., 2015; Patil et al., 2018; Yang et al., 2022). Labeling NHP TG sections with TrkB, TrkC, and Calb1 antibodies yielded inconclusive results. However, PV, which is expressed in the majority of Aβ-LTMR neurons (Usoskin et al., 2015; Yang et al., 2022), was detected in 20.1 ± 3% of NHP TG neurons (Fig. 7*D*,*H*). Although PV is also expressed in proprioceptors (Usoskin et al., 2015), it is important to note that proprioceptive neuron cell bodies in the head and neck are located in the brainstem rather than in the TG. Overall, IHC showed that NHP had peptidergic, MrgprD^+^ nonpeptidergic, TrpV1^+^, and A-LTMR-like neurons but was lacking TH as a marker for C-LTMR.

## Discussion

It is well established that distinct classes of neurons are responsible for recognizing and transmitting a variety of sensory modalities, such as heat, cold, and a variety of mechanical modes. Mechanical modalities could be detected behaviorally or by in vivo extracellular and intracellular as well as in vitro patch-clamp electrophysiological recordings. Unlike in vivo recording, when mechanical stimulus-triggered activation of sensory neurons could involve nonneuronal cells, the patch-clamp approach allows to evaluate direct activation of sensory neurons. We have used a piezoactuating device to directly apply mechanical stimuli to TG neurons. MA currents were examined in NHP TG neurons because identifying and characterizing their mechanical responses enhances the translatability of rodent studies.

This study employed only one stimulation protocol, though evidence suggests that responses vary depending on the type of mechanical stimulation used, such as stretch or vibration (Rugiero et al., 2010; Poole et al., 2014; Schaefer et al., 2023). Parameters like probe velocity, angle, and diameter, as well as tonicity of solutions, have all also been shown to influence MA current characteristics such as magnitude and kinetics (Rugiero et al., 2010; Jia et al., 2016; Verkest et al., 2022; Zeitzschel and Lechner, 2024). Patch experiments conducted under substantially uneven resting tension may also alter the kinetics of many channels (Suchyna et al., 2009). Pressure applied by the patch pipette to the membrane to form a giga seal and the size of the patch pipette could affect a cell's mechanosensitivity (Cho et al., 2002). Thus, it is possible that not all MA NHP TG neurons were appropriately assessed. In addition, the NHP TG neurons used in this study were cultured in the absence of nerve growth factor (NGF), which is a well-known sensitizer of MA currents in sensory neurons (Zeitzschel and Lechner, 2024). There is a general consensus that the protocol used in this study activates Piezo channels, which accounts for the MA currents detected in sensory neurons (Coste et al., 2010; Parpaite et al., 2021). However, Piezo2 mRNA expression in sensory neurons does not perfectly correlate with MA currents (Fernandez-Trillo et al., 2020; Parpaite et al., 2021), suggesting that the molecular mechanisms behind mechanical sensory modalities may involve some additional subunits or adaptor proteins. Whether this diversity arises from interactions with nonneuronal cells, the extracellular matrix, or neuron-specific Piezo2 complexes requires further investigation (Sekiguchi and Yamada, 2018; Parpaite et al., 2021).

We identified nine groups of NHP TG neurons based on AP characteristics and responses to mechanical stimuli (Table 1). The classification of mechanical current kinetics is well established for mouse DRG neurons (Hu and Lewin, 2006; Coste et al., 2010; Vilceanu and Stucky, 2010; Hao and Delmas, 2011). One surprising result from these experiments is a lack of clear distinctions between MA current kinetics (Fig. 2*C*). It appeared that MA currents in all, but five, NHP TG neurons had slow kinetics (>30 ms; Table 2). Strikingly, no fast MA currents were recorded from naive NHP TG neurons. While proprioceptors and A-LTMRs typically express PIEZO2 and sustain fast, rapidly adapting (RA) MA currents in mouse DRG neurons (Coste et al., 2010; Woo et al., 2015), the absence of proprioceptive cell bodies in the TG could contribute to the nonexistence of RA MA currents in NHP TG (Jerge, 1963; Lazarov, 2007). The complete absence of such currents, however, was unexpected. Notably, MA currents recorded from duck TG neurons resemble those observed in NHP TG, with the primary difference being that duck currents tend to have larger amplitudes and slower inactivation kinetics (Schneider et al., 2014, 2017). The slower inactivation kinetics in NHP neurons may suggest a mechanonociceptive function (Hao and Delmas, 2010), where slower kinetics allow for greater AP firing during mechanical stimulation (Hao and Delmas, 2010). Additionally, some studies show no difference in mechanical response threshold while others have observed higher thresholds for mechanonociceptors (Lewin and Moshourab, 2004; Viatchenko-Karpinski and Gu, 2016). Overall, while MA kinetics can be a reliable classification parameter, it is highly dependent on media for the maintenance of sensory neurons and the mechanical stimulation setup, including the piezoactuator speed and approach angle. Kinetics of MA current could also be dependent on ganglia type (TG vs DRG neurons) or species type (NHP vs rodent).

**Figure 8.**
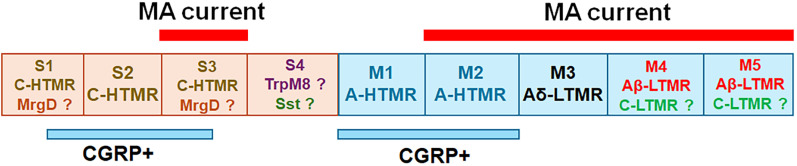
Schematic summarizing results of the study. Small-sized NHP TG neuronal groups (S1–S4) represented as the beige boxes. Medium-sized NHP TG neuronal groups (M1–M5) are the blue boxes. TG neuronal groups responding to piezoactuating mechanical simulations and exhibiting MA currents are outlined by the red bars and labeled “MA current” above each TG neuronal group. Putative CGRP^+^ groups are outlined by blue lines and labeled “CGRP^+^.” Presumed functions of the NHP TG neuronal group are indicated inside boxes.

Sensory neurons could be classified based on many parameters. Perhaps, the most thorough and detailed classifications of TG neurons could be achieved by combining single-cell RNA sequencing with electrophysiology (LaPaglia et al., 2018; Bhuiyan et al., 2024). In this regard, the information provided here may not be enough for the classification of these nine NHP TG neuronal groups. Thus, future experiments, such as patch-seq, will provide more comprehensive insights into TG neuron types (Parpaite et al., 2021). Nevertheless, certain parallels between well-characterized rodent sensory neurons and presented here NHP TG neurons could be drawn. In mouse models, DRG and TG neurons with the “bow”-shaped AP are associated with nonpeptidergic, MrgprD^+^ neurons (Patil et al., 2018; Lindquist et al., 2021). We had certain S1 neurons with “bow” shaped AP, but dV/dt did not quantify them differently from “hump” shaped AP on the falling phase. Hence, without recording from MrgprD reporter animals, it could not be conclusively defined whether some of these S1 neurons were MrgprD^+^ (Fig. 8). IHC showed that NHP TG neurons have 7.3 ± 3.1% of MrgprD^+^ neurons, which is less than reported in mouse TG (Yang et al., 2022; Bhuiyan et al., 2024) and NHP DRG (Kupari et al., 2021). Interestingly, human DRGs predominantly contain peptidergic neurons (Tavares-Ferreira et al., 2022; Jung et al., 2023; Bhuiyan et al., 2024). The S1, S2, and S3 groups displayed certain features consistent with unmyelinated (C-fiber) peptidergic neurons (Fig. 8), which has broad AP with a characteristic “hump” manifested as double peaks in a negative portion of dV/dt (Fig. 1*B,C*; Patil et al., 2018; Lindquist et al., 2021). Many of these types of DRG and TG neurons express TrpV1, which was detected in NHP TG with IHC (Fig. 7). Among the S-type of NHP TG neurons, only the S3 group showed MA currents, and their identity is not clear. Among nonmyelinated mouse neurons, MrgprD^+^ (aka IB4^+^) DRG and TG neurons are known to express PIEZO2 at higher levels and exhibit MA currents in DRG neurons (Hu and Lewin, 2006; Usoskin et al., 2015; Yang et al., 2022; Jung et al., 2023; Bhuiyan et al., 2024). APs of S4 neurons resembled those recorded from mouse DRG TH^+^ neurons (C-LTMRs). However, these neurons in DRG have prominent MA currents. Alternatively, S4 neurons could be somatostatin-positive (Sst^+^) neurons, which have been characterized by long-duration APs (Usoskin et al., 2015; Patil et al., 2018), or cooling units (TrpM8^+^), which have been previously identified in DRG neurons and display fast AHPs and shallow AHP peaks (Djouhri et al., 1998; Fang et al., 2005; Fig. 8). Notably, cooling-sensitive TG neurons, which also display fast AHPs, are rare but could also have similar action potential shape (Parpaite et al., 2021). Cold-sensing sensory neurons have been shown to be mechanically insensitive and are rare (Djouhri et al., 1998; Fang et al., 2005; Bron et al., 2014; Fernandez-Trillo et al., 2020). C-LTMR, trpM8, and Sst small groups were identified in primate DRG (Kupari et al., 2021; Jung et al., 2023).

Given the properties of M1 and M2 neurons, both groups could represent two types of A-fibers, which could be high-threshold mechanoreceptors (A-HTMR; Patil et al., 2018; Zeisel et al., 2018; Zheng et al., 2019; Sharma et al., 2020; Fig. 8). These neurons in mouse DRG and TG are CGRP^+^/trpV1^−^. Our IHC data suggest that the NHP TG is dominated by CGRP^+^/trpV1^−^ neurons (Fig. 7). Recordings also indicated a substantial presence of M1 and M2 groups (Table 1). These data imply that A-HTMRs could compose ∼30% of NHP TG neurons. Single-cell RNA sequencing from NHP DRG has identified PEP2 and PEP3 neurons, which could represent A-HTMR and may align with M1 and M2 (Kupari et al., 2021; Jung et al., 2023; Bhuiyan et al., 2024; Fig. 8). The properties of M3 neurons are similar to those of mouse DRG TrkB^+^ mouse Aδ–low-threshold mechanoreceptor (Aδ-LTMR) neurons (Usoskin et al., 2015; Patil et al., 2018), while the M4 and M5 neuron groups, with their fast APs, large MA currents with low activation thresholds, align with the properties of Aβ-LTMRs reported in the literature (Petruska et al., 2000; Patil et al., 2018; Zheng et al., 2019; Fig. 8). Since size of NHP TG neurons did not well correlate with function, M4 or M5 could also represent C-LTMR responding to stimulation large MA current (Fig. 8). Due to the absence of conduction velocity (CV) measurements in this study, we could not strictly differentiate between C, Aβ, and Aδ fibers. Moreover, Aβ fibers in TG appear more diverse than those in DRG (Harper and Lawson, 1985; Waddell and Lawson, 1990; LaPaglia et al., 2018; Okutsu et al., 2021). The relatively small population of A-LTMR neurons identified in NHP DRG through single-cell sequencing aligns with our findings (Kupari et al., 2021; Jung et al., 2023).

Overall, NHP TG neurons have many similarities with the reported properties of mouse DRG and TG neurons. However, there are notable differences such as low percentage of neurons responding to mechanical stimuli among smaller TG neurons and absence of fast and lower representation of intermediate inactivating MA currents.

## Synthesis

Reviewing Editor: Jennifer Dulin, Texas A&M University Decisions are customarily a result of the Reviewing Editor and the peer reviewers coming together and discussing their recommendations until a consensus is reached. When revisions are invited, a fact-based synthesis statement explaining their decision and outlining what is needed to prepare a revision will be listed below. The following reviewer(s) agreed to reveal their identity: Hongzhen Hu, Amanda Klein.

This is the first study to characterize electrophysiological properties of mechanically sensitive trigeminal neurons in NHPs. The reviewers agree that the study is innovative and impactful, and the data are high quality. Some additional concerns should be addressed before we can consider this manuscript for publication in eNeuro. Please see comments below.

Reviewers' comments:

Reviewer #1:

In this manuscript, the authors investigated mechanically activated currents in marmoset trigeminal ganglion (TG) neurons. They classified TG neurons into four S-type and five M-type groups based on their responsiveness to mechanical stimuli and action potential properties. They also performed immunohistochemistry to confirm the presence of known neuronal markers, including TRPV1, CGRP, MrgprD, TH, and PV, in marmoset TG neurons. This is the first study investigating mechanically activated currents in TG neurons derived from non-human primates, making it highly innovative. I have the following comments.

1. Whether mechanically activated currents in marmoset TG neurons can be inhibited by mechanosensitive ion channel inhibitors such as GsMTx4 will be an interesting question.

2. Since previous studies have reported that the osmotic pressure of the external and internal solutions in patch-clamp recordings significantly affects the amplitude and inactivation kinetics of mechanically activated currents, it is essential to include this information in the Methods section.

3. The authors analyzed and compared electrophysiological properties and mechanically activated currents, including cell capacitance and maximum mechanically activated currents. Maximum current density can also provide valuable information; therefore, it would be beneficial to include it in the results. This value can be easily obtained by dividing the maximum current by cell capacitance.

4. In the immunostaining results, a pan-neuronal marker antibody, such as PGP9.5 or TUBB3, should be used to label all neurons.

Reviewer #2:

The manuscript entitled, "Investigating Mechanically Activated Currents from Trigeminal Neurons of Non-Human Primates", attempts to investigate the properties of mechanically activated (MA) neurons in the trigeminal ganglia (TG) of non-human primates (marmosets). The paper has clear methodology and the results are easily digestible, but there are several key points that the authors should discuss to increase the impact of their findings:

1. Can the authors explain where the "S-" and "M-type" nomenclature comes from when designating TG neuron subtypes?

2. Table 1 does an excellent job of breaking down each neuron subtype based on electrophysiological properties. Were any cut-offs determined for each of the parameters? For example, if one was recording from a M4 or M5 subtype, the AHP parameter is clearly the most distinct, but at what point would this neuron be considered one over the other? Maybe a flow-chart would be helpful for readers if attempted in their own labs.

3. The description of each neuron subtype, along with potential candidates for involvement in a class of nerve fibers these might contribute (e.g. M1 and M2 as high-threshold mechanoreceptors, A-HTMR) is a very interesting point made in the discussion. This information as a supplemental table would be useful - along with other parameters (e.g potential TRPM8 sensitivity) would help to tie the ephys data with the IHC data.

4. What do the blue arrows in panels 7G and 7H refer to? Were MrgD and TrpV1 analyzed for co-expression?

5. Was any attempt to segregate the TG based on innervation area (e.g. V1 vs V2 vs V3)?

6. The following citations may help with the discussion to compare with human TG: Bhuiyan et al., Science Advances, 2024; Jung et al., Nature Communications, 2023; LaPaglia et al., Cephalagia, 2017
